# Draft Genome Sequence of *Altererythrobacter troitsensis* JCM 17037, Isolated from the Sea Urchin *Strongylocentrotus intermedius*

**DOI:** 10.1128/genomeA.01556-15

**Published:** 2016-01-14

**Authors:** Peng Zhou, Yue-Hong Wu, Hong Cheng, Chun-Sheng Wang, Xue-Wei Xu

**Affiliations:** Laboratory of Marine Ecosystem and Biogeochemistry, Second Institute of Oceanography, State Oceanic Administration, Hangzhou, China

## Abstract

The habitats of the genus *Altererythrobacter* are various, including marine sediment, seawater, rhizosphere of wild rice, desert sand, etc. The genome of the type strain of *Altererythrobacter troitsensis* JCM 17037, isolated from sea urchin, was sequenced. This study would not only facilitate the understanding of the physiology, adaptation, and evolution of the *Altererythrobacter* species, but also provide a good resource for the study of synthesis of astaxanthin, since several enzymes involved in the production of astaxanthin were predicted.

## GENOME ANNOUNCEMENT

The genus *Altererythrobacter* was proposed by Kwon et al. (1). It accommodates bacteria inhabiting various habitats, such as marine sediment (1, 2), seawater (3–5), tidal flats (6), the rhizosphere of wild rice (7), desert sand (8), etc. Interestingly, *Altererythrobacter troitsensis* JCM 17037 was isolated from the sea urchin *Strongylocentrotus intermedius* (9). Here, we present the draft genome of *A. troitsensis* JCM 17037 to facilitate the study of its physiology, adaptation, and evolution.

Genomic DNA was extracted with the AxyPrep Bacterial Genomic DNA Miniprep Kit (Corning Life Sciences, USA), and then was sequenced using Illumina HiSeq2000 platform (Novogene Bioinformatics Technology Co. Ltd., Beijing). A 500-bp paired-end library was constructed. The sequencing generated 710 M of clean data, representing ~245-fold genome coverage. Reads were *de novo* assembled into contigs and subsequently joined into scaffolds using SOAPdenovo (version 2.0.1) (10) and Abyss (version 1.5.2) (11). The assembly k-mer was tested from 57 to 64 for seeking the optimal value of *k* = 59 using Abyss. MUMmer was used to estimate the assembly quality (12).

The draft genome of *A. troitsensis* JCM 17037 comprises 2,902,267 bp, with an average G+C content of 64.7%, consisting of 9 contigs. The draft genome was annotated by using RAST (13). The draft genome sequence contains 2,847 candidate CDSs. In addition, 45 tRNA genes and 3 rRNA genes were identified. The genome of *A. troitsensis* JCM 17037 would not only facilitate the study of the physiology, adaptation, and evolution of the *Altererythrobacter* species but may also provide a good resource for further study on the predicted enzymes, such as epoxide hydrolase, alkane hydroxylase, β-carotene hydroxylase, and β-carotene ketolase involved in the production of astaxanthin.

### Nucleotide sequence accession numbers.

This whole-genome shotgun project has been deposited at DDBJ/EMBL/GenBank under the accession number LMAU00000000. The version described in this paper is version LMAU01000000.
